# The curious case of exploding quantum dots: anomalous migration and growth behaviors of Ge under Si oxidation

**DOI:** 10.1186/1556-276X-8-192

**Published:** 2013-04-25

**Authors:** Ching-Chi Wang, Po-Hsiang Liao, Ming-Hao Kuo, Tom George, Pei-Wen Li

**Affiliations:** 1 Department of Electrical Engineering, National Central University, Chung-Li 320, Taiwan; 2 Center for Nano Science and Technology, National Central University, Chung-Li 320, Taiwan; 3 Zyomed Corporation, Altadena, California 91001, USA

**Keywords:** Germanium quantum-dot, Migration, Oxidation

## Abstract

We have previously demonstrated the unique migration behavior of Ge quantum dots (QDs) through Si_3_N_4_ layers during high-temperature oxidation. Penetration of these QDs into the underlying Si substrate however, leads to a completely different behavior: the Ge QDs ‘explode,’ regressing back almost to their origins as individual Ge nuclei as formed during the oxidation of the original nanopatterned SiGe structures used for their generation. A kinetics-based model is proposed to explain the anomalous migration behavior and morphology changes of the Ge QDs based on the Si flux generated during the oxidation of Si-containing layers.

## Background

Since their inception in the early 1980s [1], quantum dots (QDs) have found a widespread application in advanced electronics, photonics, memories, thermoelectrics, metrology, and biosensing devices [2-6]. For semiconductor QDs, the key challenge for the production of these mostly self-assembled nanostructures is to achieve precise control over the formation of QDs of desired sizes at specific locations and targeted depths of penetration within an embedding matrix. Our group has successfully demonstrated a unique approach to deliberately locate Ge QDs of desired sizes, locations, and depths within Si-based semiconductor nanostructures using the control available through lithographic nanopatterning and selective oxidation of the nanopatterned Si_1-*x*_Ge_*x*_ layers [7-10].

**Figure 1:**
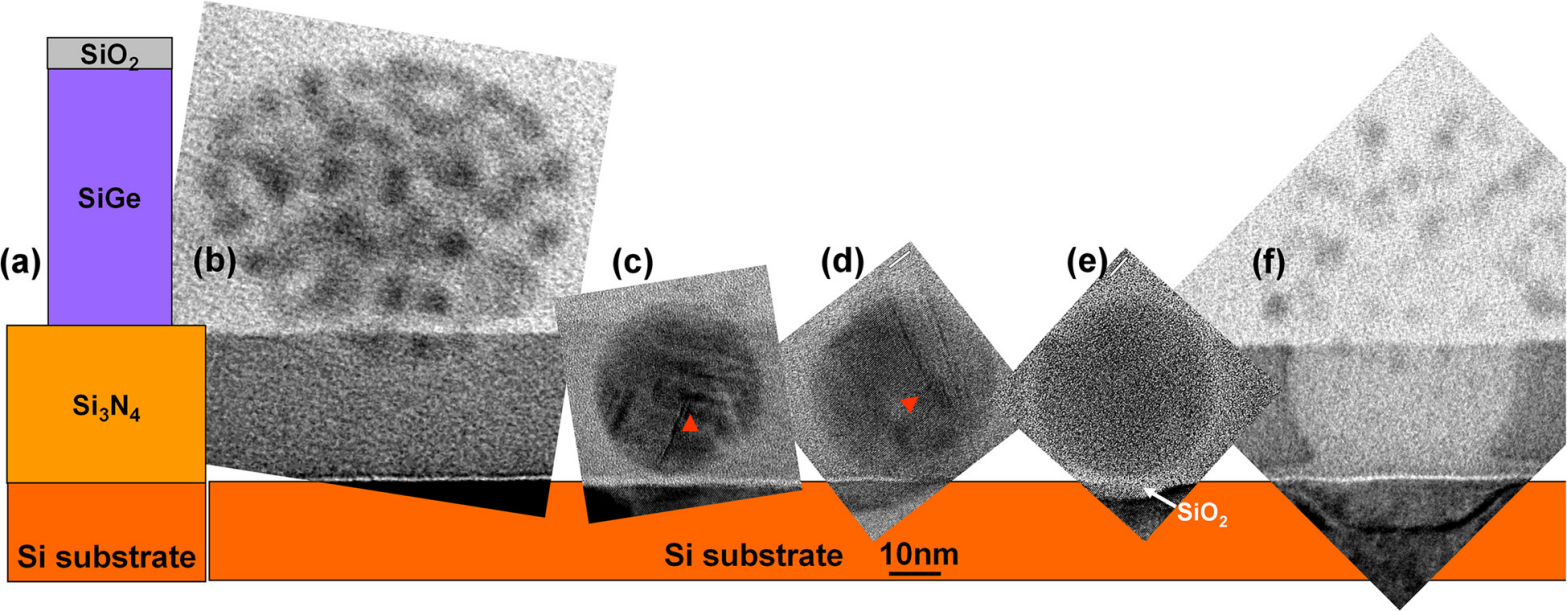
**Oxidation time evolution of 30-nm Ge QDs.** (**a**) Schematic of the SiO_2_/SiGe/Si_3_N_4_ pillar over the Si substrate before oxidation. CTEM images illustrating the time evolution of 30-nm Ge QDs formed after thermal oxidation of Si_0.85_Ge_0.15_ pillars of 50-nm diameter for (**b**) 25, (**c**) 35, (**d**), 60, (**e**) 75, and (**f**) 90 min, respectively. Arrows in (**c**) and (**d**) highlight the presence of stacking faults and twins within the QDs. Micrographs (**b**) to (**f**) are all at the same magnification.

During the course of our work, we have previously demonstrated [7,8,11] the ability to produce large, spherical Ge QDs derived from smaller Ge nuclei generated in the as-oxidized Si_1-*x*_Ge_*x*_ layers. The QD growth occurs via Ostwald ripening [12,13] during a unique ‘burrowing’ process. In this process, a few of these nuclei grow in size as they migrate through an underlying Si_3_N_4_ buffer layer [See Figure 1c]. This interesting phenomenon also results in the change in morphology of the originally irregularly shaped Ge nuclei to the more ideal and theoretically predicted [14] spherical shape observed for the large Ge QDs without any preferred crystallographic faceting. We have explained the migration behavior as due to the burrowing Ge QDs catalytically enhancing the local oxidation of the Si_3_N_4_ buffer layer [9]. The Si_3_N_4_ dissociates to release Si atoms that migrate to the QD. Subsequently, the Si diffuses to the distal end of the QD to be oxidized to form SiO_2_ thus facilitating the deeper penetration of the QD into the Si_3_N_4_ layer. The high crystalline quality and high purity of the spherical Ge QDs was confirmed by high-resolution cross-sectional transmission electron microscopy (CTEM) and electron dispersive X-ray spectroscopy (EDX) measurements, as well as by the significantly reduced dark current and greatly improved long-wavelength (1,550 nm) responsivity of photodetectors fabricated from these Ge QD/Si heterostructures [10].

Given the remarkable, experimentally observed property of Ge QDs to ‘divine’ the presence of Si-bearing layers by preferentially migrating towards them, we decided to investigate this effect further by continuing the high-temperature oxidation process (Figure 1) to allow the spherical Ge QDs to ‘transit’ through the Si_3_N_4_ buffer layer and penetrate the pure Si substrate below (Figure 1c,d,e). However, when the Ge QD burrows through the Si_3_N_4_ buffer layer and encounters the Si substrate, a completely different phenomenon is observed (Figure 1f): the original spherical QD, instead of growing larger, ‘explodes’ into smaller Ge fragments that now appear to migrate away from the Si substrate with further oxidation. In a sense, this new behavior is parallel to the fantasy story, ‘The Curious Case of Benjamin Button,’ [15] in which, with the passing of time, Button, rather than aging, instead regresses back to his early childhood. In a similar fashion, the large, spherical QDs appear to regress back to their origins as many smaller, irregularly shaped QDs originally generated within the as-oxidized Si_1-*x*_Ge_*x*_ layers.

## Methods

The nominal QD fabrication process involves the tri-layer growth of 15- to 30-nm-thick Si_3_N_4_/20- to 60-nm-thick polycrystalline Si_0.85_Ge_0.15_/5-nm-thick SiO_2_ using low-pressure chemical vapor deposition over a Si substrate. The topmost, thin SiO_2_, layer is deposited as a hard mask for the subsequent plasma etching for producing the lithographically-patterned SiGe nanopillars. The SiO_2_ cap also prevents the evaporation of Ge during the final, high-temperature oxidation process for generating Ge QDs from the original SiGe layers. Using a combination of electron-beam lithography and SF_6_/C_4_F_8_ plasma patterning processes, SiGe nanopillar structures of various sizes (50- to 100-nm widths) were fabricated and then subjected to thermal oxidation at 900°C for 35 to 90 min in an H_2_O ambient for generating the Ge QDs. Oxidation times vary based on the thickness of the nanopillars. It takes between 5 and 25 min at 900°C within the H_2_O ambient to completely oxidize polycrystalline Si_0.85_Ge_0.15_ pillars that are between 20- and 60-nm thick and convert them into Ge crystallites.

CTEM, scanning transmission electron microscopy (STEM), and EDX were conducted using a JEOL JEM-2100 LaB6 transmission electron microscope (JEOL, Akishima-shi, Japan) and a FEI Tecnai Osiris transmission electron microscope (FEI, Hillsboro, OR, USA). Great care was taken to prepare clean TEM samples with no surface contamination. Additionally, STEM observations were conducted under conditions (200 KV and beam current of 100 μA) of minimal radiation-induced damage to the Ge QDs.

## Results and discussion

### Ge QDs in SiO_2_ matrix

The oxidation of each SiGe nanopillar proceeds radially inwards in an anisotropic manner and preferentially converts the Si within the pillar into SiO_2_, while squeezing the Ge released from solid solution within each poly SiGe grain into an irregular-shaped Ge crystallite that ostensibly assumes the crystal orientation and a portion of the morphology of the original poly SiGe grain (Figure 1b). Thus, within this newly formed SiO_2_ matrix, Ge nuclei, 5 to 7 nm in size, appear in a self-assembled cluster with random morphology and crystalline orientation. Further oxidation results in the observed Ostwald ripening behavior with some of the nuclei in proximity to the Si_3_N_4_ buffer layer growing at the expense of the other previously formed Ge nuclei. Additionally, as described previously, the Ostwald ripening and the overall change in morphology to a more spherical shape occur as a consequence of the Ge QD burrowing into the underlying Si_3_N_4_ buffer layer (Figure 1c,d,e).

### Ge QDs in Si_3_N_4_ matrix

The Ge QD migrates through the underlying Si_3_N_4_ layer in a two-step catalytic process, during which the QD first enhances the local decomposition of the Si_3_N_4_ layer, releasing Si that subsequently migrates to the QD. In the second step, the Si rapidly diffuses through the QD, perhaps interstitially [16-20], and is ultimately oxidized at the distal surface of the QD, generating the SiO_2_ layer above the QD. It takes approximately 75 min for a 30-nm diameter Ge QD (Figure 1e) to transit through the 30-nm-thick Si_3_N_4_ layer and make contact with the Si substrate below.

### Ge QDs encountering the Si substrate

**Figure 2:**
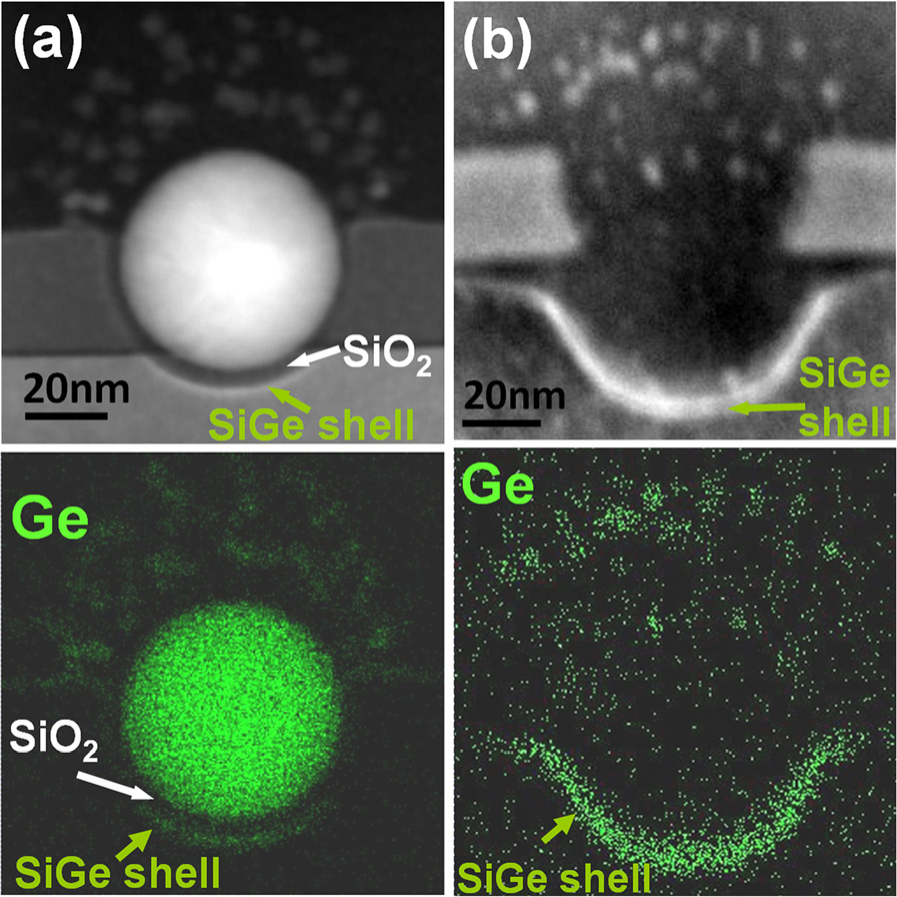
**STEM and EDX images of 50-nm Ge QDs formed after thermal oxidation of Si**_**0.85**_**Ge**_**0.15 **_**pillars.** Si_0.85_Ge_0.15_ pillars with a diameter of 100 nm were thermally oxidized at 900°C for (**a**) 60 and (**b**) 90 min.

Dramatic changes in the QD morphology and shape occur when the Ge QD encounters the Si substrate after penetrating through the Si_3_N_4_ buffer layer following a longer duration (90 min) oxidation process (Figure 1f). We discovered two new phenomena: first, the Ge QD and the Si substrate are separated by a thin layer of SiO_2_ that is not only conformal with the QD but also conformal with a cup-shaped depression that appears to be ‘scooped out’ of the Si substrate (Figures 1f and 2a). Further examination of the edges of the cup with CTEM-EDX mapping reveals that it is ‘lined’ with Ge (Figure 2a). Second, with further oxidation, the Ge QD appears to explode into a number of smaller Ge ‘dew drops’ that appear to migrate away from the Si substrate (Figure 2b). The Ge dew drops are about 5 to 7 nm in size, similar in size to the Ge nuclei formed in the as-oxidized SiGe nanopillars described in the ‘Ge QDs in SiO_2_ matrix’ section above.

Thus, we have shown that the Ge QD exhibits two distinct types of morphological and migrational behaviors depending on whether it encounters a Si_3_N_4_ layer or the Si substrate. As mentioned above, in a previous paper [9], we have provided a detailed explanation for the behavior of Ge QDs penetrating Si_3_N_4_ buffer layers. In this paper, we propose a new explanation for the radically different behavior of the very same QDs now interacting with the Si substrate. Here, we draw parallels from previous studies on the oxidation rate of silicon showing a marked dependence of the oxidation on the Ge content in Si and the oxygen flux [21-25].

**Figure 3:**
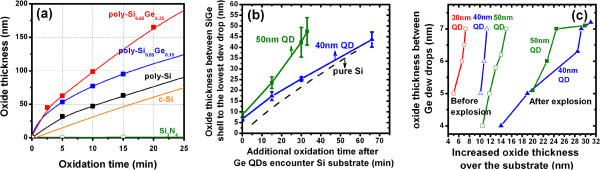
**Growth kinetics of poly-Si**_**1-*x***_**Ge**_***x ***_**oxidation and migration characteristics of Ge drew drops.** (**a**) Growth kinetics of polycrystalline Si_1-*x*_Ge_*x*_, single-crystalline Si, and Si_3_N_4_ oxidation at 900°C in H_2_O ambient. (**b**) The oxide thickness between the SiGe shell and the bottom of the lowest Ge dew drop as a function of additional oxidation time after Ge QDs encountering Si substrate. (**c**) The oxide thickness between the Ge dew drops as a function of the increased thickness of the oxide layer over the Si substrate. The error bars were determined by the extensive observation on more than 25 QDs for each data point.

We begin by considering the two steps in which these changes occur in the migration and morphology of the Ge QDs. The two steps are the following:

a. **SiGe ‘Shell’ formation**: Upon ‘contact’ with the Si substrate, i.e., with a thin oxide separating the QD from the substrate, it becomes thermodynamically and kinetically favorable for Ge atoms to migrate from the QD and dissolve within the Si substrate to form a thin, cup-shaped SiGe alloy shell (Figure 2). This is because of the release of the free energy of mixing for the SiGe alloy [26,27]. Assuming that the kinetics of Ge diffusion and alloying with Si is not rate-limiting at these high oxidation temperatures, we propose that the thickness (4 to 5 nm; observed from STEM and EDX images in Figure 2) and composition (40% to 60%; derived from the enhanced oxidation rate to be discussed below) of the SiGe shell is essentially determined by an equilibrium established between the lowering of free energy due to SiGe alloy formation while simultaneously accompanied by an increase in free energy due to the lattice strain experienced by the SiGe shell formed on the Si substrate [27]. This thin SiGe shell formed on the Si substrate surface also plays a pivotal role in the very different behavior of the Ge QD during further oxidation. Unlike in the case of the Si_3_N_4_ oxidation, where no such SiGe surface layer exists, the SiGe shell is experimentally observed to significantly enhance the oxidation rate of the Si substrate by as much as 2 to 2.5 times. Figure 3a shows our experimental data for the oxidation kinetics of polycrystalline Si_1-*x*_Ge_*x*_ layers in an H_2_O ambient at 900°C. The enhancement in the oxidation rate of polycrystalline Si_1-*x*_Ge_*x*_ as a function of Ge composition appears to be well approximated by 1 + *ax*, where the enhancement factor *a* ranges from 2.5 to 3.05 and *x* is the mole fraction of Ge in a Si_1-*x*_Ge_*x*_ alloy. The enhancement factor for polycrystalline Si_1-*x*_Ge_*x*_ oxidation is very close to the previous results which report an enhancement factor of 2 to 4 for the oxidation of single crystalline Si_1-*x*_Ge_*x*_ layers over that for Si [21-23]. Using this relationship, we estimate the Ge content of our thin SiGe shell to be between 40% and 60%. In contrast to the Ge QD-enhanced oxidation of the Si_3_N_4_ buffer layers, where a nearly constant, approximately 2.5-nm thickness of SiO_2_ exists between the burrowing QD and the Si_3_N_4_ interface, the oxide thickness between the QD and the Si substrate (or between the SiGe shell and the bottom of the lowest Ge dew drop) appears to increase with time and follows the expected oxidation kinetics of SiGe layers (Figure 3b).

In the case of the Si_3_N_4_ oxidation, we proposed that the 2.5-nm oxide thickness separating the QD from the nitride was essentially determined by a dynamic equilibrium that exists between the concentration of Si atoms generated from the dissociation of the Si_3_N_4_ and the oxygen flux [9]. The bulk of the Si atoms generated by the Si_3_N_4_ dissociation is consumed in generating SiO_2_ behind the Ge QD and thereby facilitating the burrowing process.

Just as in the case of Si_3_N_4_ layer oxidation [9,10], the oxidation of the Si substrate also results in the generation of fluxes of Si atoms which migrate to the Ge QD. However, the flux of Si atoms generated by the Si substrate is far greater than the corresponding flux generated by the decomposition of Si_3_N_4_ layers [28]. A very large volume expansion occurs during both Si and Si_3_N_4_ oxidations. The volume occupied by the SiO_2_ is larger by about a factor of 2.2 than the volume occupied by the pure silicon substrate used to form the SiO_2_, whereas the expansion factor for the case of Si_3_N_4_ oxidation is about 1.64 [29]. Also, as we have previously presented [9,10], most of the oxide that is generated in the case of the Si_3_N_4_ oxidation occurs behind the burrowing QD and thus does not affect the morphology of the migrating QD. In the case of the Si substrate penetration however, the oxidation mediated by the thin SiGe shell results in very large compressive stresses in the growing oxide layer and corresponding tensile stresses in the silicon substrate in the near surface region. The oxidation-generated stress results in the generation of Si interstitials according to the following equation [28]:

$$\left(1+2\gamma \right)\mathrm{Si}+2O_{I}+2\mathrm{\beta V}↔\mathrm{Si}O_{2}+2\mathrm{\gamma I}+\mathrm{stress}.$$

where *γ* is the mole fraction of Si interstitials generated during the oxidation process, and *β* is the mole fraction of Si vacancies (*V*). *O*_I_ represents the mole fraction of oxygen atoms which diffuse interstitially to oxidize the silicon, and *I* denotes the mole fraction of Si interstitials. A stress term is included because it is unlikely that the point defects alone could relieve all of the stress generated by the volume expansion. It is generally agreed that Si interstitials generated during Si oxidation diffuse into the growing oxide instead of diffusing into the silicon substrate. These are then the Si interstitials that subsequently migrate towards the Ge QD. Thus, two completely different effects occur based just on the magnitude of the Si flux. In the low flux case (Si_3_N_4_ layer oxidation), the dominant site for the Si oxidation is the distal end of the QD. In contrast, oxidation of the Si substrate enhanced by the thin SiGe shell results in the generation of a significantly larger flux of Si interstitials [16-18,28]. As opposed to the nitride oxidation mechanism, the high Si flux makes it possible for oxidation to occur simultaneously at a number of additional sites namely, not just at the Si substrate surface but also within the QD itself.

b. **QD explosion**: The higher Si atom fluxes appear to cause heterogeneous defect sites within the QD to now become ‘activated’ as new and additional sites for silicon oxidation. Proof for our proposed mechanism above can be derived, by analogy, from previous works on the dependence of Si oxidation on oxygen flux [25,30]. It has been shown previously that the oxidation rate is indeed linearly dependent on oxygen flux, with the pre-factor term of the oxidation-kinetics equation being enhanced by the increased oxygen concentration. According to the Deal-Groove model [25], oxide thickness increases with oxidation time per the equation: *x*_0_^2^ + *Ax*_0_ = *B*(*t* + *τ*), where *τ* corresponds to a shift in the time coordinate which corrects for the presence of the initial oxide layer. The parabolic rate constant *B* = 2*D*_*eff*_C^*^/*N*_1_ is proportional to the equilibrium concentration of the oxygen flux (C^*^), which has been experimentally demonstrated to be proportional to the partial pressure of the oxidizing species in the gaseous ambient [30]. *D*_*eff *_is the effective diffusion coefficient, and *N*_1_ is the number of oxygen molecules incorporated per unit volume of the oxide layer. The coefficient *A* is independent of the partial pressure, leading to the linear rate constant *B/A* which linearly increases with oxygen flux as well.

In a similar manner, we propose that the higher Si fluxes being generated via substrate oxidation now make it possible for higher rates of oxidation to occur at heterogeneous defect sites including stacking faults and twins within the QD (Figure 1c,d) and hence cause it to ‘explode’ into multiple Ge fragments, almost identical in size to the as-oxidized Ge islands formed from the original SiGe nanopillars. With further silicon dioxide generation, the Ge ‘dew drops’ subsequently migrate outward, from the core of the original monolithic Ge QD from which they came with increasing time through the increase in the thickness of the SiO_2_ layers separating them. Eventually, Si atom diffusion from the substrate to the dew drops slows down as the oxide thickness between them and the substrate increases. This decreased supply of Si atoms results in the oxide layers between the dewdrops achieving a limiting thickness of 4 to 8 nm (Figure 3c).

## Conclusion

We have observed the unique and anomalous phenomenon of completely different Ge QD growth and migration behaviors within Si_3_N_4_ layers versus within the Si substrate during high-temperature oxidation. The Ge migration behavior and morphology change appears to be directly dependent on the Si flux generated during the oxidation of Si-containing layers. When the flux of Si is low (as in the case of the Si_3_N_4_), the Ge migrates as a large, spherical QD that grows at the expense of smaller Ge nuclei. In contrast, when the Si flux is high, as in the oxidation of the Si substrate (enhanced by the formation of a thin SiGe shell), internal defect sites within the QD become activated as sites for Si oxidation, causing QD to explode and almost regress to its origins as smaller separated Ge nuclei.

## Competing interests

The authors declare that they have no competing interests.

## Authors' contributions

CW carried out the TEM experimentation and analysis. PL and MK carried out the Ge QD growth and kinetics analysis. TG conceived the mechanism of Ge QD explosion and drafted the manuscript. PL conceived the study, supervised the work, contributed to data analysis and the manuscript preparation. All authors read and approved the final manuscript.
